# Rural-urban differences in prevalence of and risk factors for refractive errors among school children and adolescents aged 6–18 years in Dalian, China

**DOI:** 10.3389/fpubh.2022.917781

**Published:** 2022-08-29

**Authors:** Yachen Wang, Lei Liu, Zhili Lu, Yiyin Qu, Xianlong Ren, Jiaojiao Wang, Yan Lu, Wei Liang, Yue Xin, Nan Zhang, Lin Jin, Lijing Wang, Jian Song, Jian Yu, Lijun Zhao, Xiang Ma, Lijun Zhang

**Affiliations:** ^1^Department of Ophthalmology, The Third People's Hospital of Dalian, Dalian, China; ^2^Department of Ophthalmology, Dalian Third People's Hospital Affiliated to Dalian Medical University, Dalian, China; ^3^Department of Ophthalmology, Guangdong Eye Institute, Guangdong Provincial People's Hospital, Guangzhou, China; ^4^Department of Ophthalmology, The First Affiliated Hospital of Dalian Medical University, Dalian, China; ^5^He Eye Hospital, Dalian, China; ^6^Beijing Center for Diseases Prevention and Control, Beijing, China

**Keywords:** prevalence, refractive errors, risk factors, schoolchildren, urban-rural disparity

## Abstract

**Purpose:**

To assess the prevalence of refractive errors (REs) in school children aged 6–18 years in urban and rural settings in Dalian, Northeast of China.

**Methods:**

This is a school-based cross-sectional survey using multi-stage randomization technique. Six- to eighteen-year-old school children from elementary schools, junior and senior high schools from a rural area and an urban area in Dalian were included in December 2018. All subjects underwent a comprehensive questionnaire and eye examination.

**Results:**

A total of 4,522 school children with 6–18 years of age were investigated. The age, gender-adjusted prevalence of myopia, and anisometropia were 82.71 and 7.27% among the urban students as compared to 71.76% and 5.41% among the rural ones (OR = 1.80, 95 % CI = 1.53 - 2.11, *P* < 0.001; OR = 1.29, 95 % CI = 1.00–1.67, *P* = 0.049), respectively. The hyperopia was less common in urban students than in rural ones (5.63 *vs*. 10.21%; OR = 0.54, 95 % CI: 0.43–0.67, *P* < 0.001). However, there was no significant difference in prevalence of astigmatism between urban (46.07%) and rural (44.69%) participants (OR = 0.96, 95 % CI: 0.84–1.10, *P* = 0.559). The differences on prevalence of REs were attributed to different social-demographic and physiologic factors.

**Conclusions:**

The students from urban settings are more likely to have myopia and anisometropia but less likely to have hyperopia than their rural counterparts. Although considerable attention had been paid to controlling REs, it is necessary to further consider the urban-rural differences in REs.

## Introduction

The refractive errors (REs), especially myopia, have become the primary cause of vision impairment (VI) and preventable blindness in children. Myopia suggests a significant increase in prevalence globally in the past 50 years and become a significant public health problem across the globe, especially in East Asian countries like Singapore and China (1, 2). In Singapore, the incidence of myopia in children is about 62% (3), and Chinese having higher rates compared with Indians and Malays (4). In Guangzhou China, the prevalence of myopia among schoolchildren is 49.7%, much higher than the United States (20%) (5), Australia (11.9%) (6), and Nigeria (1.9%) (7). Among them, the increase in school children is particularly remarkable, 73.9–90% of high school students were myopia in urban areas of Aisa (8–10).

Various factors, including genetic and environmental factors play a role in the etiology of myopia. Genetically, the prevalence of myopia in children is greater if their parents are myopic (11, 12). Rapidly changing environmental factors are predominant in determining the current patterns of myopia (13). Near-work, time outdoors have shown to be associated with the occurrence and development of myopia (14, 15). Region of habitation, are also thought to influence presence of REs (16). In China, there were two population-based studies reported the rural-urban prevalence of REs among children and adolescents. According to Beijing Pediatric Eye Study, first, they found that prevalence of myopia was significantly associated with urban region (17). However, in further analysis on factors for myopia, prevalence of myopia was not significantly associated with urban region of habitation after adjusting for age, gender, school type, family income, and parental myopia (18). Although separate prevalence rates of REs in rural vs. urban children were reported in Shandong Children Eye Study (19), there remain some gaps in this study. Those prevalence rates were crude rates without adjusting age, gender and other potential confounders.

At present, the prevalence and risk factors of REs in urban and rural school children in Northeast China are still unclear. Whether there are differences in the associated factors of REs in different regions needs further research. In order to understand the prevalence of REs and the risk factors disparity among school children (6–18 years old) between urban and rural settings, we performed this school-based survey in the urban and rural areas of Dalian, Northeast China.

## Methods

### Participants

This school-based study was initially performed in December 2018. Multi-stage random cluster approach was conducted for sampling. In the first step, one district from each of the rural (Wafangdian County) and urban (Xigang District) regions of Dalian was randomly selected. In the second step, one primary school, one junior high school, and one senior high school were randomly selected from each of the two selected districts. In the third step, two classes of each grade were of randomly selected from each of the including schools. In the final step, all students of the selected classes with age of 6–18 years were sampled. The exclusion criteria were the followings: (1) Participants who reported eye conditions within the last month (e.g., optical correction with othokeratology, eye injuries, conjunctivitis, and corneal irritation); (2) Participants whose parents refused to sign the informed consents.

The study was approved by the respective ethics committees of The Third People‘s Hospital of Dalian and the Health commission of Dalian. This study followed the Declaration of Helsinki. Written consent was obtained from the parents of all children and teenagers.

### Interview and data collection

In current study, all participants and their parents completed a detailed questionnaire form. The quality of the questionnaire was controlled by head teacher in each class. The questionnaires are conducted at home.

The questionnaire included two parts (participants' information section and parents' information section). Basic socio-demographic data, such as age, gender, ethnic origin, habitation in urban or rural areas, degree of class and grade, and medical history was included in the first part of the questionnaire. Moreover, this questionnaire section additionally included questions on near-work activities such as the amount of time spent on studying or watching television, mobile phone and on computer activities per day. The first part of the questionnaire also includes questions about outdoor activities such as how long the children spent in outdoor activities per day. The first part of the questionnaire was filled in by the children and assisted by their parents. For very young children who could not read or understand the questionnaire very well (e.g., the youngest children of 6 years old), help was sought from their parents.

In the second part, information of parents' education level, refractive error history (e.g., myopia, hyperopia, astigmatism) were obtained using questionnaire from participants‘ parents.

After the interview, comprehensive ophthalmological examinations were conducted on the school premises by two trained optometrists. Refractive error measurements, uncorrected visual acuity as well as best corrected visual acuity (BCVA) were tested using non-cycloplegic auto-refractometry (AR-1, NIDEK, Japan) by a senior experienced optometrist. Moreover, intraocular pressure (IOP) was measured by non-contact tonometry (NT-510, NIDEK, Japan). Axial length was tested by IOL Master (Carl Zeiss Meditec, Jena, Germany). The mean of three readings were taken.

### Measured variables

The definitions of refractive error vary across the selected prevalence studies, we choose the definition that is common in clinical use (19, 20). The spherical equivalent (SE) of the refraction was calculated as the spherical refractive error plus half of the minus cylindrical refractive error. Myopia was defined as SE < −0.5 dioptres (D), and hyperopia was defined as SE > +0.5 D in one or both eyes. Myopia can be classified as low, moderate, or high myopia. Low myopia was defined as SE −0.75 D to −2.75 D, moderate myopia was defined as SE −3.00 D to −4.75 D, and high myopia was defined as SE ≤ -5.0 D. Astigmatism was defined as ≥ +0.75 D of the cylinder in either eye. Anisometropia was defined as difference between right eye to left eye in refractive error (SE) of ≥1.0 D.

### Statistics

The data were analyzed using a commercially available statistical program SAS 9.3 (SAS institute, Cary, NC, USA) and SPSS 20.0 (SPSS Inc., Chicago, IL). Only the data for eye with high severity of refraction is presented. Participants included in the final analysis were divided into three age groups (6–10, 11–15, and 16–18 years old, respectively) which were consistent with the distribution of education level for including participants. The age- and gender-specific prevalence rates of REs and subtypes were assessed. The difference of the variables (age groups, gender and region) with REs was assessed using the Student's *t*-test for the continuous variables and the Pearson's χ^2^ test for the categorical variables. Logistic regression analysis was performed to determine risk factors using odds ratio (OR) estimates with 95% confidence intervals (CI). A multivariate regression analysis was performed with *P*-value < 0.05 being required for entering the model. All *P*-values were 2-sided and considered statistically significant when <0.05.

## Results

In our study, the seven schools had a total student of 4,583 individuals 6–18 years old, and all of participants were given offers to accept the body and eye examination. In total, 4,522 students (2,336 boys) participated in the all examination, corresponding to an overall response rate of 98.7% (98.9% for urban and 98.4% for rural, respectively). Socio-demographic characteristics were compared between urban students and rural students (Table 1). The rural students and the urban students group varied significantly in the level of age with a significantly higher frequency of 6–10 and 11–15 years old in the rural students, and complementarily, a significantly higher frequency of 16–18 years old in the urban students. The urban students were more likely to be with higher frequency of parental refractive error (*P* < 0.001), annual household income exceeds Renminbi (RMB) 200,000 Yuan ($ 28,982 USD) (*P* < 0.001), higher parental education level (*P* < 0.001), daily hours of outdoor activities ≤ 2 h (*P* < 0.001), and have higher level of height, weight as well as BMI (*P* < 0.001) but lower refractive status (*P* < 0.001) than those rural students. Further, there is no significant difference in gender (*P* = 0.062), and daily hours of near-work (*P* = 0.981) distribution between urban and rural students.

**Table 1:** Characteristics of all participants.

**Variables**	**Overall**	**Urban**	**Rural**	***P***
*N* (subjects, %)	4,522	2,429 (53.72)	2,093 (46.28)	
Gender				0.062
Male (%)	2,336 (51.66)	1,286 (52.94)	1,050 (50.17)	
Female (%)	2,186 (48.34)	1,143 (47.06)	1,043 (49.83)	
Age (year)				<0.001
6–10	1,708 (37.77)	856 (35.24)	852 (40.71)	
11–15	1,797 (39.74)	855 (35.20)	942 (45.00)	
16–18	1,017 (22.49)	714 (29.56)	299 (14.29)	
Height (cm)	151.28 ± 19.67	152.97 ± 19.95	149.26 ± 19.12	<0.001
Weight (cm)	45.82 ± 18.96	46.98 ± 19.48	44.44 ± 18.18	<0.001
BMI (kg/m^2^)	19.18 ± 4.60	19.19 ± 4.64	19.15 ± 4.56	<0.001
Parental refractive error (%)				<0.001
Myopia	1,580 (34.94)	918 (37.79)	662 (31.63)	
No Myopia	2,942 (65.06)	1,511 (62.21)	1,431 (68.37)	
Parental education level (%)				<0.001
Junior high school	1,649 (36.47)	574 (23.63)	1,075 (51.36)	
Senior high school	1,286 (28.44)	668 (27.50)	618 (29.53)	
Bachelor	1,365 (30.19)	986 (40.59)	379 (18.11)	
Master	222 (4.90)	201 (8.28)	21 (1.00)	
Annual household income (yuan, %)				<0.001
>200,000	504 (11.15)	373 (15.36)	131 (6.26)	
≤ 200,000	4,018 (88.85)	2,056 (84.64)	1,962 (93.74)	
Daily hours of near-work (%)				0.981
<2 h	331 (7.32)	178 (7.33)	153 (7.31)	
≥2 h	4,191 (92.68)	2,251 (92.67)	1,940 (92.69)	
Daily hours of outdoor activities (%)				<0.001
>2 h	2,790 (61.70)	1,348 (55.50)	1,442 (68.90)	
≤ 2 h	1,732 (38.30)	1,081 (44.50)	651 (31.10)	
Spherical equivalence (D)	−2.18 ± 3.30	−2.53 ± 2.57	–1.76 ± 3.96	<0.001

Crude and adjusted-prevalence of REs distributed by region is shown in Table 2. There were differences in the prevalence of different REs values between the urban and rural students. After adjusted for age and gender, the prevalence of overall myopia, low myopia, moderate myopia and high myopia among urban students was 82.71, 70.52, 50.88, and 38.68%, and it was higher than that among rural students (71.76, 58.54, 32.16, and 21.55%, respectively). Similar results were found in anisometropia, the age, gender-standardized prevalence of anisometropia was higher in the urban students than in the rural students (7.27 *vs*. 5.41%, *P* = 0.013). However, the age, gender-adjusted prevalence of hyperopia in the rural students was higher than that in the urban students (10.21 *vs*. 5.63%, *P* < 0.001). Additionally, no difference was found in astigmatism between the rural and urban students (44.69 *vs*. 46.07%, *P* = 0.379).

**Table 2:** Crude and age-adjusted prevalence of different refractive errors.

**Items**	***N***	**Crude prevalence (95%CI)**	**Age, gender-adjusted prevalence (95%CI)**	***P***
Myopia				<0.001
Urban	1,840	75.75% (74.01%, 77.42%)	82.71% (80.89%, 84.38%)	
Rural	1,297	61.97% (59.78%, 63.95%)	71.76% (69.24%, 74.15%)	
Over all	3,137	69.37% (68.01%, 70.70%)	–	
Low myopia				<0.001
Urban	930	61.22% (58.78%, 63.68%)	70.52% (67.76%, 73.13%)	
Rural	801	50.16% (47.61%, 52.52%)	58.54% (55.34%, 61.67%)	
Over all	1,731	55.55% (53.80%, 57.29%)	–	
Moderate myopia				<0.001
Urban	494	45.61% (42.68%, 48.62%)	50.88% (46.65%, 55.09%)	
Rural	296	27.11% (24.51%, 29.78%)	32.16% (28.25%, 36.34%)	
Over all	790	36.32% (34.33%, 38.37%)		
High myopia				<0.001
Urban	416	41.39% (38.31%, 44.41%)	38.68% (33.73%, 43.87%)	
Rural	200	20.08% (17.65%, 22.62%)	21.55% (17.78%, 25.87%)	
Over all	616	30.78% (28.80%, 32.84%)	–	
Hyperopia				<0.001
Urban	178	7.33% (6.40%, 8.50%)	5.63% (4.78%, 6.63%)	
Rural	286	13.66% (12.26%, 15.20%)	10.21% (8.85, 11.75%)	
Over all	464	10.26% (9.41%, 11.18%)	–	
Astigmatism				0.379
Urban	1,107	45.57% (43.53%, 47.50%)	46.07% (44.00%, 48.15%)	
Rural	847	40.47% (38.37%, 42.58%)	44.69% (42.37%, 47.02%)	
Over all	1,954	43.21% (41.77%, 44.66%)	–	
Anisometropia				0.013
Urban	196	8.07% (7.02%, 9.19%)	7.27% (6.27%, 8.42%)	
Rural	111	5.30% (4.44%, 6.37%)	5.41% (4.47%, 6.54%)	
Over all	307	6.79% (6.09%, 7.56%)	–	

CI, Confidence Interval.

Bivariate analysis showed factors associated with REs among all subjects (Table 3). Currently, students‘ age, region of habitation, average parental refractive error, parental education level, annual household income and daily hours of near-work were associated with myopia (all *P* < 0.05). However, students‘ age, region of habitation, average parental refractive error, annual household income and daily hours of near-work were associated with hyperopia (all *P* < 0.05). Further, students‘ age, gender, region of habitation, average parental refractive error, parental education level and daily hours of near-work were associated with astigmatism (all *P* < 0.05). In addition, there was a significant correlation between age, region of habitation, annual household income and anisometropia (all *P* < 0.05).

**Table 3:** Bivariate regression results for both the urban and rural participants.

	**Myopia**		**Hyperopia**		**Astigmatism**		**Anisometropia**	
	**OR (95%CI)**	***P***	**OR (95%CI)**	***P***	**OR (95%CI)**	***P***	**OR (95%CI)**	***P***
Region
Rural	1		1		1		1	
Urban	1.97 (1.73, 2.24)	<0.001	0.50 (0.41, 0.61)	<0.001	1.24 (1.10, 1.40)	<0.001	1.61 (1.27, 2.04)	<0.001
Gender
Male	1		1		1		1	
Female	1.10 (0.97, 1.25)	0.143	0.94 (0.78, 1.14)	0.550	0.79 (0.70, 0.89)	<0.001	0.97 (0.77, 1.22)	0.790
Age
6–10	1		1		1		1	
11–15	7.07 (6.05, 8.27)	<0.001	0.20 (0.16, 0.26)	<0.001	1.81 (1.57, 2.08)	<0.001	2.88 (2.06, 4.03)	<0.001
16–18	18.86 (14.57, 24.43)	<0.001	0.19 (0.14, 0.26)	<0.001	3.92 (3.33, 4.62)	<0.001	4.67 (3.31, 6.59)	<0.001
Average parental refractive error
Without	1		1		1		1	
With	1.24 (1.09, 1.42)	0.002	0.76 (0.62, 0.93)	0.009	1.34 (1.19, 1.51)	<0.001	1.10 (0.87, 1.39)	0.438
Annual household income (yuan)
>200,000	1		1		1		1	
≤ 200,000	0.73 (0.59, 0.91)	0.006	1.79 (1.21, 2.64)	0.003	0.88 (0.72, 1.06)	0.170	0.68 (0.48, 0.94)	0.020
Daily hours of near-work
<2 h	1		1		1		1	
≥2 h	4.76 (3.75, 6.04)	<0.001	0.28 (0.21, 0.37)	<0.001	1.86 (1.45, 2.37)	<0.001	1.55 (0.91, 2.64)	0.105
Daily hours of outdoor activities
>2 h	1		1		1		1	
≤ 2 h	0.93 (0.81, 1.06)	0.249	0.90 (0.74, 1.10)	0.309	1.05 (0.93, 1.18)	0.455	1.10 (0.87, 1.39)	0.451
Parental education level
Middle school	1		1		1		1	
High school	1.25 (1.06, 1.47)	0.007	1.09 (0.86, 1.39)	0.464	1.28 (1.10, 1.48)	0.001	1.17 (0.87, 1.57)	0.304
Bachelor	1.14 (0.98, 1.34)	0.097	0.93 (0.73, 1.19)	0.566	1.28 (1.11, 1.49)	0.001	1.25 (0.94, 1.67)	0.122
Master	0.95 (0.70, 1.29)	0.756	0.57 (0.32, 1.02)	0.057	0.95 (0.71, 1.27)	0.721	0.82 (0.43, 1.56)	0.544

OR, odds ratio; CI, Confidence Interval.

Stepwise multiple logistic models were used to analyze the correlation between region and REs (Table 4). In model 1, after controlling for age and gender, the risk of students living in urban setting developing myopia, hyperopia, and anisometropia were 1.88 (95%CI: 1.62–2.18, *P* < 0.001), 0.53 (95%CI: 0.43–0.65, *P* < 0.001), 1.07 (95%CI: 0.94–1.21, *P* = 0.300), and 1.37 (95%CI: 1.07–1.76, *P* = 0.013), respectively. However, there is no significant difference on presence of astigmatism between students in rural and urban settings (OR: 1.07, 95%CI: 0.94–1.21, *P* = 0.300). In model 2 adjusting with age, gender and any variables analyzed significantly in the bivariate analysis (Table 3), students living in urban were 1.80 (95% CI: 1.53–2.11, *P* < 0.001) times more likely to be myopic, 1.29 (95% CI: 1.00, 1.67, *P* = 0.049) times more likely to be anisometropia, but 0.54 (95% CI: 0.43–0.67, *P* < 0.001) times less likely to be hyperopia. Further, there was no significant association between the student's area of residence and astigmatism (OR: 0.96, 95%CI: 0.84–1.10, *P* = 0.559).

**Table 4:** Multivariate regression results for the differences on the risk for REs by region of habitation.

		**Myopia**		**Hyperopia**		**Astigmatism**		**Anisometropia**	
		**OR (95%CI)**	***P***	**OR (95%CI)**	***P***	**OR (95%CI)**	***P***	**OR(95%CI)**	***P***
Model 1	Rural	1		1		1		1	
	Urban	1.88 (1.62, 2.18)	<0.001	0.53 (0.43, 0.65)	<0.001	1.07 (0.94, 1.21)	0.300	1.37 (1.07, 1.76)	0.013
Model 2	Rural	1		1		1		1	
	Urban	1.80 (1.53, 2.11)	<0.001	0.54 (0.43, 0.67)	<0.001	0.96 (0.84, 1.10)	0.559	1.29 (1.00, 1.67)	0.049

Model 1: adjusted with age and gender.

Model 2: adjusted with age, gender and any the variables analyzed significantly in the bivariate analysis.

OR, odds ratio; CI, Confidence Interval.

Bivariate and multivariate analyses identified the risk factors for the presence of any REs in the urban (Supplementary Table S1) and rural (Supplementary Table S2) students. After multivariate analysis, increasing age and daily hours of near-work were found to be a risk factor for myopia in both the rural and urban groups but with parental refractive error was a risk factor for myopia only in the rural group (all *P* < 0.001). Increasing age, daily hours of near-work and lower annual household income were independent risk factors for hyperopia in the urban participants while increasing age, daily hours of near-work and average parental refractive error were independent risk factors for hyperopia in the rural participants (all *P* < 0.05). Female gender was found to be reduced risk for astigmatism in the urban population (*P* < 0.001). Further, increasing age (*P* < 0.001) and average parental refractive error (*P* < 0.05) were independent risk factors for astigmatism in the urban participants. However, increasing age, higher level of annual household income, parental education level and with average parental refractive error were independent risk factors for astigmatism in the rural participants (all *P* < 0.001).

For anisometropia, we found that only increasing age was a risk factor for urban students but both increasing age and higher level of annual household income were risk factor anisometropia in the rural students (all *P* < 0.05).

## Discussion

Currently, our study provides the population-based cross-sectional data on the region-specific prevalence of REs and its associated risk factors among the urban and rural school children and adolescents across several gradients of age groups which have different socio-cultural factors in Northeast China. First, our findings revealed that the students living in urban setting have higher prevalence rates and risk for myopia and anisometropia than students living in rural, while the prevalence and risk of hyperopia in the urban students was lower than that in the rural students. Moreover, there is no difference on the prevalence and risk of astigmatism between the urban and rural students. Secondly, the prevalence disparities of REs may be due to the various factors between rural and urban areas. Thirdly, there appeared to be significant difference in factors of REs between study participants residing in urban and rural settings.

Consistent with our findings, a previous meta-analysis reported that children from urban environments have 2.6 times the odds of myopia compared with those from rural environments (16). Similarly, the prevalence of myopia in urban setting was higher compared with rural setting based in other region of China (Shandong and Guangzhou) (19, 21). In southern China, the prevalence of myopia in urban children was 73.1% (15 years old), while the prevalence of myopia in rural children was 36.8% (13 years old) and 53.9% (17 years old) (21, 22). However, this disparity was did not adjusted comprehensive variables. Furthermore, we found studies regarding the other prevalence of REs between urban and rural students in China are limited. Table 5 shows the comparison of prevalence of REs between rural and urban settings among school children and adolescents in mainland China. The crude prevalence of myopia, hyperopia, astigmatism, and anisometropia in urban ranges from 5 to 87.7%, 1 to 35.9%, 2.0 to 42.7%, and 7.9%, while in rural ranges from 13.75 to 60%, 1 to 49.2%, 3.75 to 32.1%, and 6.1%, respectively. Our prevalence astigmatism was higher than the surveys while prevalence of myopia, hyperopia and anisometropia data fall somewhere in between. Further, the prevalence disparity of astigmatism between rural and urban areas is still controversial. In Shandong children eye study, students with urban habitation had higher prevalence of astigmatism (40.7%) than those with rural habitation (32.1%) (19). In Dezful County of Iran, school children with urban habitation also had higher prevalence of astigmatism (21%) than those with rural habitation (14.8%) (38). The varying difference in the prevalence of REs between rural and urban habitation may be attributed to the different living environments and variability in the cut-off point adopted to define the presence of REs. In Shandong study, the generational REs shift was measured to by cycloplegia while in our study without cycloplegia (19).

**Table 5:** Comparison of the reported prevalence of refractive errors in selected population-based studies in school children and adolescents in mainland China.

**References**	**Area**	**Sample size**	**Survey year**	**Age rage (years)**	**Study area**	**Definition for REs**	**Prevalence of myopia**	**Prevalence of hyperopia**	**Prevalence of astigmatism**	**Prevalence of Anisometropia**
Wu et al. (23)	12 cities*	43,771	N.A	11.45 ± 2.65	R+U	Questionnaires	25.7% (R) *vs*. 38% (U)	N.A	N.A	N.A
He et al. (24)	Yangxi	2,400	2005	13–17	R	Myopia SE ≤ -0.50 D; Hyperopia SE > +2.00 D; Astigmatism: cylinder of > or = 0.75 D (Cycloplegia)	33.0	1.0	25.3	N.A
Wu et al. (19)	Shandong^#^	6,026	2013	4–18	R+U	Myopia: SE ≤ −0.50 D; Mild Hyperopia +0.5D < SE ≤ +2.0 D; Medium to Marked Hyperopia SE > +2.0 D; Astigmatism: cylindrical RE ≥ 0.75 D; Anisometropia: difference between right eye to left eye in SE of ≥ 1.0 D (Cycloplegia)	30.7 (R) vs 43.5 (U)^†^	Mild: 49.2 (R) vs. 35.9 (U)^†^; Medium to Marked: 6.4 (R) vs. 5.2 (U)^†^	32.1 (R) vs. 40.7 (U)^†^	6.1 (R) vs. 7.9 (U)^†^
You et al. (17)	Beijing	15,066	N.A.	7–18	R+U	SE ≤ −0.50 D (Cycloplegia)	64.9 (overall)	NA	NA	NA
Pan et al. (25)	Mojiang	4,778		7.7–13.8	R	SE < −0.5 D (Cycloplegia)	29.4% (7.7 y) 2.4% (13.8 y)	NA	NA	NA
Congdon et al. (26)	Xichang	1,892	2007	11.4–17.1	U	SE < −0.5 D (Cycloplegic)	62.3%	NA	NA	NA
Guo et al. (27)	Ejina	1,565	2012	6–21	R	SE ≤ −0.50 D (Cycloplegic)	60.0%	NA	NA	NA
He et al. (21)	Guanghzou	4,364	2002–2003	5–15	U	Myopia SE ≤ −0.50 D; Hyperopia SE > +2.00 D; Astigmatism: cylinder of > or = 0.75 D (autorefraction under cycloplegia)	78.4	1%	42.7%	NA
Li et al. (28)	Anyang	4,861	2011	5–16	U	Myopia SE ≤ −0.50 D; Hyperopia SE > +2.00 D; (Cycloplegia)	3.9% (5–6 y); 67.3% (15- 16 y)	23.3% (grade 1); 1.2% (grade 7)	NA	NA
Li et al. (29)	Heilongjiang	1,675	2008–2009	5–18	U	Myopia SE ≤ −0.50 D; Hyperopia SE > +0.50 D; Astigmatism: cylinder of > or = 0.75 D (Cycloplegia)	5.0%	1.6%	2.0%	N.A
Chen et al. (30)	Fenghua	43,858	2001–2015	17–18	U	Myopia SE ≤ −0.50 D; (Without Cycloplegia)	79.5% (2001); 87.7% (2015)	NA	NA	NA
Sun et al. (31)	Qingdao	3,753	2015–2016	10–15	U	Myopia SE < −0.50 D (Cycloplegia)	52.02%	NA	NA	NA
Guo et al. (32)	Beijing	35,745	2016	6–18	R+U	Myopia SE < −0.50 D (Without cycloplegia)	70.9%	NA	NA	NA
Ma et al. (33)	Shanghai	8,267	2013	10	U	Myopia SE ≤ −0.50 D; Hyperopia SE > +0.50 D; (Cycloplegia)	52.2%	2.6%	NA	NA
Guo et al. (34)	Guangzhou	3,055	2014	6–15	U	Myopia SE ≤ −0.50 D; (Cycloplegia)	47.3%	NA	NA	NA
Lyu et al. (35)	Beijing	4,249	2011	5–14	U	Myopia SE ≤ −0.50 D; (Cycloplegia)	36.7%	NA	NA	NA
Wu et al. (36)	Beijing	4,677	N.A.	16–18	U	Myopia SE ≤ −1.00 D; (Without cycloplegia)	80.7%	NA	NA	NA
Pi et al. (37)	Yongchuan	3,070	2006–2007	6–15	R	Myopia SE ≤ −0.50 D; Hyperopia SE ≥+2.00 D; Astigmatism: cylinder of > or = 1.0 D (Cycloplegia)	13.75%	3.26%	3.75%	NA

^*^Beijing, Shaoxing, Shenzhen, Chongqing, Guizhou, Taiyuan, Ma'anshan, Shenyang, Urumqi, Changsha, Yinchuan and Zhengzhou.

^#^Weihai (urban), Guanxian (rural).

^†^Statistical significant, P < 0.05.

REs, refractive errors; U, urban; R, rural; SE, spherical equivalence; D, dioptres; NA, not applicable.

To date, there are few studies investigating the different associated factors for REs between rural and urban participants. The current study performed in Dalian reports the effect and possible factors on REs in a wide age range among the Chinese urban and rural students. For both rural and urban students, we found that increasing age and longer daily hours of near-work were independently associated with myopia, which was consistent with previous reports in Beijing urban students (39, 40). However, in another rural study (Handan), there was no significant association between daily near work and presence of myopia even adjusted with potential confounders (22). Interestingly, significant association between parental refractive error and myopia was found in rural participants rather than urban individuals. Further, myopia was associated with senior high parental education among our rural participants which was consistent with Yangxi County eye study (24). In addition, increasing age and longer daily hours of near-work were protective factors for hyperopia in both rural and urban students. Moreover, the prevalence rates of hyperopia for urban students were higher with lower annual household income which was consistent with study outcomes among adults in Sumatra, Indonesia (41). In both rural and urban children and adolescents, risk factors that were related to astigmatism were age and parental refractive error. Interestingly, female gender has lower risk for astigmatism among urban students and lower annual household income level has lower risk for astigmatism among rural ones. In study in Singaporean children (7–9 years), girls had significantly greater progression of astigmatism than did boys (42) which was inconsistent with our findings. Increasing age was an independent risk factor for anisometropia in both rural and urban participants. Protective factor, only in the rural arm, was lower annual household income level. These socio-demographic and lifestyle factors disparities may contribute to the prevalence disparities of REs between rural and urban students in this study.

A noteworthy finding is that the prevalence of REs including myopia was not significant with daily hours of outdoor activities in both urban and rural children and adolescents. In contrast, outdoor activities are negatively associated with myopia after adjustment for potential factors in both urban regions such as Hubei (43) and Qingdao (31)and rural settings e.g., Handan (22). Three cross section studies [mainland China (44), Taiwan (45) and Singapore (46)] did not find any relationship between outdoor activities and presence of myopia.

The major strength of this study included a comprehensive population-based sample from a large city, urban and rural areas; reasonable response rates; and reliable demographic data. This data are extremely useful for healthcare providers to develop long-term strategies to combat avoidable visual impairments due to REs. It is heartening to see a declining prevalence of REs as compared to epidemiological studies done in past worldwide. The study also found socio-demographic and health-related factors for REs between rural and urban students. It is possible that modulating this variable may control the occurrence of RE, however, this warrants longitudinal studies. A limitation of the study is the inability to validate the causal relationship between the significant risk factors and presence of REs. Cohort studies are recommended for the future. In addition, we excluded those participants with optical correction using othokeratology which may lower-evaluating the prevalence of myopia.

## Conclusions

Our study investigated the overall prevalence of REs including myopia, hyperopia, astigmatism and anisometropia in rural and urban areas in Dalian China in children and adolescents aged 6–18 years. The students with urban habitation had a higher prevalence of myopia and anisometropia but a lower risk of hyperopia than those with rural habitation. With multivariate logistic regression, the factors regarding REs between rural and urban participants were different. Herein, the implementation and findings from this screen will guide the efficient prevention strategy of refractive error and eye care services in urban and rural school children and adolescents.

## Data availability statement

The raw data supporting the conclusions of this article will be made available by the authors, without undue reservation.

## Ethics statement

The studies involving human participants were reviewed and approved by the Ethics Committee of Dalian Third People's Hospital. Written informed consent to participate in this study was provided by the participants' legal guardian/next of kin.

## Author contributions

LZhan, LL, XM, JW, and WL contributed to the conception of the study. YW, ZL, YQ, JW, YL, WL, YX, NZ, LJ, LW, JS, JY, and LZhao performed the experiment. YW, LL, XR, and LZhan contributed significantly to analysis and manuscript preparation. YW, LL, and XR performed the data analyses and wrote the manuscript. LZhan, LL, and XM helped perform the analysis with constructive discussions. All authors contributed to the article and approved the submitted version.

## Funding

This study was supported by Dalian Health Commission Department; Ministry of Education of Dalian. The funders had no role in study design, data collection and analysis, decision to publish, or preparation of the manuscript.

## Conflict of interest

The authors declare that the research was conducted in the absence of any commercial or financial relationships that could be construed as a potential conflict of interest.

## Publisher's note

All claims expressed in this article are solely those of the authors and do not necessarily represent those of their affiliated organizations, or those of the publisher, the editors and the reviewers. Any product that may be evaluated in this article, or claim that may be made by its manufacturer, is not guaranteed or endorsed by the publisher.
