# Supplementary material for: Rural-urban differences in prevalence of and risk factors for refractive errors among school children and adolescents aged 6–18 years in Dalian, China
Source: Front Public Health. 2022 Aug 29;10:917781. doi: 10.3389/fpubh.2022.917781 (PMC9465045; doi:10.3389/fpubh.2022.917781)
Supplement: Supplementary file 1 [file Data_Sheet_1.pdf]

Table S1. Bivariate and multivariate regression results for REs in urban participants.

	Myopia				Hyperopia				Astigmatism				Anisometropia			
	**bivariate**		Multivariate*		**bivariate**		Multivariate*		**bivariate**		Multivariate*		**bivariate**		Multivariate*	
	OR(95%CI)	P	OR(95%CI)	P	OR(95%CI)	P	OR(95%CI)	P	OR(95%CI)	P	OR(95%CI)	P	OR(95%CI)	P	OR(95%CI)	P
Gender																
Male	1		1		1		1		1		1		1		1	
Female	1.05(0.87,1.26)	0.611	1.07(0.86,1.34)	0.546	0.83(0.61,1.13)	0.230	0.86(0.62,1.19)	0.365	**0.73(0.62,0.85)**	**<0.001**	**0.73(0.61,0.86)**	**<0.001**	0.87(0.65,1.16)	0.343	0.92(0.69,1.24)	0.600
Age (year)																
6 - 10	1		1		1		1		1		1		1		1	
11 - 15	**9.54(7.35,12.40)**	**<0.001**	**9.04(6.86,11.91)**	**<0.001**	**0.23(0.15,0.34)**	**<0.001**	**0.21(0.13,0.34)**	**<0.001**	**2.04(1.67,2.49)**	**<0.001**	**2.10(1.71,2.58)**	**<0.001**	**2.74(1.77,4.25)**	**<0.001**	**2.74(1.76,4.25)**	**<0.001**
16 - 18	**11.18(8.34,14.99)**	**<0.001**	**10.58(7.85,14.75)**	**<0.001**	**0.32(0.21,0.47)**	**<0.001**	**0.35(0.23,0.54)**	**<0.001**	**3.94(3.19,4.87)**	**<0.001**	**4.05(3.26,5.04)**	**<0.001**	**4.07(2.65,6.27)**	**<0.001**	**4.07(2.64,6.26)**	**<0.001**
Average parental refractive error																
Without	1		1		1		1		1		1		1		1	
With	1.00(0.83,1.21)	0.983	0.94(0.83,1.07)	0.332	0.85(0.62,1.17)	0.317	0.90(0.64,1.27)	0.560	**1.25(1.06,1.47)**	**0.008**	**1.29(1.08,1.54)**	**0.005**	1.01(0.75,1.36)	0.942	0.96(0.70,1.30)	0.781
Daily hours of near-work																
< 2h	1		1		1		1		1		1		1		-	
≥2h	**4.94(3.58,6.80)**	**<0.001**	**1.99(1.38,2.85)**	**<0.001**	**0.27(0.18,0.41)**	**<0.001**	**0.44(0.8,0.68)**	**<0.001**	1.94(1.39,2.72)	<0.001	1.22(0.84,1.78)	0.287	1.31(0.70,2.46)	0.401	-	-
Daily hours of outdoor activities																
＞2h	1		1		1		1		1		1		1		1	
≤ 2h	0.86(0.72,1.04)	0.127	-	-	0.98(0.72,1.33)	0.892	-	-	0.91(0.77,1.07)	0.243	-	-	0.99(0.74,1.32)	0.922	-	-
Annual household income (yuan)																
>200,000	1		1		1		1		1		1		1		1	
≤ 200,000	0.89(0.68,1.17)	0.411	-	-	**2.04(1.17,3.57)**	**0.012**	**2.75(1.50,5.06)**	**0.001**	1.12(0.89,1.41)	0.318	-	-	0.85(0.57,1.26)	0.409	-	-
Parental education level																
Junior high school	1		1		1		1		1		1		1		1	

Senior high school	0.84(0.64,1.11)	0.221	1.00(0.72,1.38)	0.986		1.26(0.82,1.95)	0.291	-	-		1.02(0.81,1.27)	0.890	1.02(0.80,1.30)	0.883		0.84(0.57,1.24)	0.381	-	-
Bachelor	0.64(0.50,0.83)	0.001	0.94(0.70,1.28)	0.695		1.31(0.87,1.96)	0.190	-	-		0.97(0.79,1.19)	0.748	1.11(0.88,1.39)	0.397		0.82(0.57,1.18)	0.293	-	-
Master	0.50(0.35,0.73)	<0.001	0.76(0.48,1.19)	0.231		0.73(0.34,1.54)	0.403	-	-		0.71(0.51,0.99)	0.042	0.85(0.59,1.23)	0.384		0.60(0.31,1.17)	0.131	-	-

*Adjusted with age, gender, and any the variables analysed significantly in the bivariate analysis and potential confounders

Table S2. Bivariate and multivariate regression results for REs in rural participants.

	Myopia					Hyperopia					Astigmatism					Anisometropia			
	**bivariate**		Multivariate*			**bivariate**		Multivariate*			**bivariate**		Multivariate*			**bivariate**		Multivariate*	
	OR(95%CI)	P	OR(95%CI)	P		OR(95%CI)	P	OR(95%CI)	P		OR(95%CI)	P	OR(95%CI)	P		OR(95%CI)	P	OR(95%CI)	P
Gender																			
Male	1		1			1		1			1		1			1		1	
Female	1.20(1.00,1.43)	0.045	1.16(0.93,1.44)	0.197		0.99(0.77,1.27)	0.919	1.07(0.82,1.40)	0.623		0.89(0.75,1.06)	0.200	0.84(0.69,1.01)	0.101		1.27(0.86,1.86)	0.230	1.28(0.86,1.91)	0.226
Age (years)																			
6 - 10	1		1			1		1			1		1			1		1	
11 - 15	**6.60(5.36,8.12)**	**<0.001**	**6.39(5.13,7.96)**	**<0.001**		**0.18(0.13,0.25)**	**<0.001**	**0.19(0.14,0.26)**	**<0.001**		**1.62(1.33,1.97)**	**<0.001**	**1.60(1.29,1.97)**	**<0.001**		**3.10(1.84,5.22)**	**<0.001**	**3.31(1.89,5.80)**	**<0.001**
16 - 18	**57.60(30.18,109.94)**	**<0.001**	**53.84(28.08,103.22)**	**<0.001**		**0.11(0.06,0.21)**	**<0.001**	**0.12(0.06,0.22)**	**<0.001**		**3.96(3.00,5.23)**	**<0.001**	**3.83(2.86,5.12)**	**<0.001**		**4.89(2.71,8.83)**	**<0.001**	**4.82(2.57,9.03)**	**<0.001**
Average parental refractive error																			
Without	1		1			1		1			1		1			1		1	
With	**1.44(1.18,1.74)**	**<0.001**	**1.66(1.30,2.13)**	**<0.001**		**0.75(0.56,0.99)**	**0.041**	**0.68(0.50,0.91)**	**0.010**		**1.43(1.19,1.72)**	**<0.001**	**1.31(1.06,1.61)**	**<0.001**		1.24(0.83,1.85)	0.287	1.20(0.80,1.81)	0.383
Daily hours of near-work																			
＜2h	1		1			1		1			1		1			1		1	
≥2h	**5.07(3.48,7.38)**	**<0.001**	**2.34(1.52,3.59)**	**<0.001**		**0.27(0.19,0.40)**	**<0.001**	**0.52(0.35,0.78)**	**0.001**		1.69(1.17,2.43)	0.005	1.31(0.86,1.98)	0.206		2.13(0.77,5.86)	0.144	-	-
Daily hours of outdoor activities																			
＞2h	1		1			1		1			1		1			1		1	
≤ 2h	**0.82(0.68,1.00)**	**0.048**	0.94(0.75,1.19)	0.622		1.00(0.76,1.31)	0.996	-	-		1.19(0.99,1.44)	0.070	-	-		1.18(0.78,1.76)	0.436	-	-

Annual household income (yuan)																			
＞200,000	1		1			1		1			1		1			1		1	
≤ 200,000	068(0.45,1.01)	0.057	-	-		1.19(0.67,2.10)	0.559	-	-		**0.54(0.37,0.78)**	**0.001**	**0.65(0.44,0.97)**	**0.035**		**0.45(0.25,0.83)**	**0.011**	**0.53(0.28,0.98)**	**0.043**
Parental education level																			
Junior high school	1		1			1		1			1		1			1		1	
Senior high school	**1.28(1.04,1.58)**	**0.022**	**1.52(1.17,1.97)**	**0.002**		1.20(0.90,1.62)	0.220	-	-		**1.42(1.15,1.74)**	**0.001**	**1.35(1.08,1.68)**	**0.009**		1.42(0.90,2.23)	0.128	-	-
Bachelor	1.15(0.90,1.47)	0.275	1.15(0.84,1.56)	0.377		1.09(0.77,1.56)	0.623	-	-		**1.58(1.24,2.02)**	**<0.001**	**1.49(1.14,1.95)**	**0.004**		1.64(0.99,2.73)	0.054	-	-
Master	0.66(0.27,1.59)	0.349	0.59(0.20,1.75)	0.341		1.77(0.58,5.37)	0.316	-	-		1.15(0.47,2.85)	0.755	1.14(0.44,2.97)	0.782		-	-	-	-

*Adjusted with age, gender, and any the variables analysed significantly in the bivariate analysi
